# Cooperative bi-exponential decay of dye emission coupled via plasmons

**DOI:** 10.1038/s41598-018-27901-4

**Published:** 2018-06-22

**Authors:** David P. Lyvers, Mojtaba Moazzezi, Vashista C. de Silva, Dean P. Brown, Augustine M. Urbas, Yuri V. Rostovtsev, Vladimir P. Drachev

**Affiliations:** 1 Department of Physics and Advanced Materials Manufacturing Processing Institute, University of North Texas, Denton, TX 76203 USA; 2 Skolkovo Institute of Science and Technology, Moscow, 121205 Russia; 3 Air Force Research Laboratory, 3005 Hobson Way, Wright Patterson AFB, Ohio, OH 45433 USA; 4 UES, Inc., 4401 Dayton-Xenia Rd, Dayton, OH 45432 USA

## Abstract

Bi-exponential decay of dye fluorescence near the surface of plasmonic metamaterials and core-shell nanoparticles is shown to be an intrinsic property of the coupled system. Indeed, the Dicke, cooperative states involve two groups of transitions: super-radiant, from the most excited to the ground states and sub-radiant, which cannot reach the ground state. The relaxation in the sub-radiant system occurs mainly due to the interaction with the plasmon modes. Our theory shows that the relaxation leads to the population of the sub-radiant states by dephasing the super-radiant Dicke states giving rise to the bi-exponential decay in agreement with the experiments. We use a set of metamaterial samples consisting of gratings of paired silver nanostrips coated with Rh800 dye molecules, having resonances in the same spectral range. The bi-exponential decay is demonstrated for Au\SiO_2_\ATTO655 core-shell nanoparticles as well, which persists even when averaging over a broad range of the coupling parameter.

## Introduction

Collective spontaneous emission is theoretically and experimentally investigated for an inhomogeneously broadened ensemble of emitters coupled to a metamaterial mode in one case and core-shell structure in the second instance. It was previously predicted with Monte Carlo numerical methods that a fully inverted inhomogeneously broadened ensemble of *N* two-level systems coupled to a single-mode low-*Q* cavity possess intrinsically bi-exponential emission dynamics^1^. If all atoms are initially inverted, the first spontaneously emitted photons trigger a buildup of a large macroscopic atomic polarization during the emission of a super-fluorescent pulse. The peak intensity and emission rate of such a super-fluorescent pulse is ~*N* times larger compared to that of *N* independent atoms^2^, which in a classical picture corresponds to parallel dipole moments of all radiating atoms. However, there also exist the so-called subradiant states, in which the energy remains partially trapped in the atomic system since some of the atomic dipoles become antiparallel and the macroscopic polarization of the system is zero; i.e., they cannot couple to a radiation field. Numerical simulations by Temnov and Woggon^1^ shows that a strong inhomogeneous broadening in the Dicke model leads to an intrinsically bi-exponential temporal evolution of the emission dynamics in which a fast superradiant decay is followed by a slow subradiant energy relaxation.

**Figure 1:**
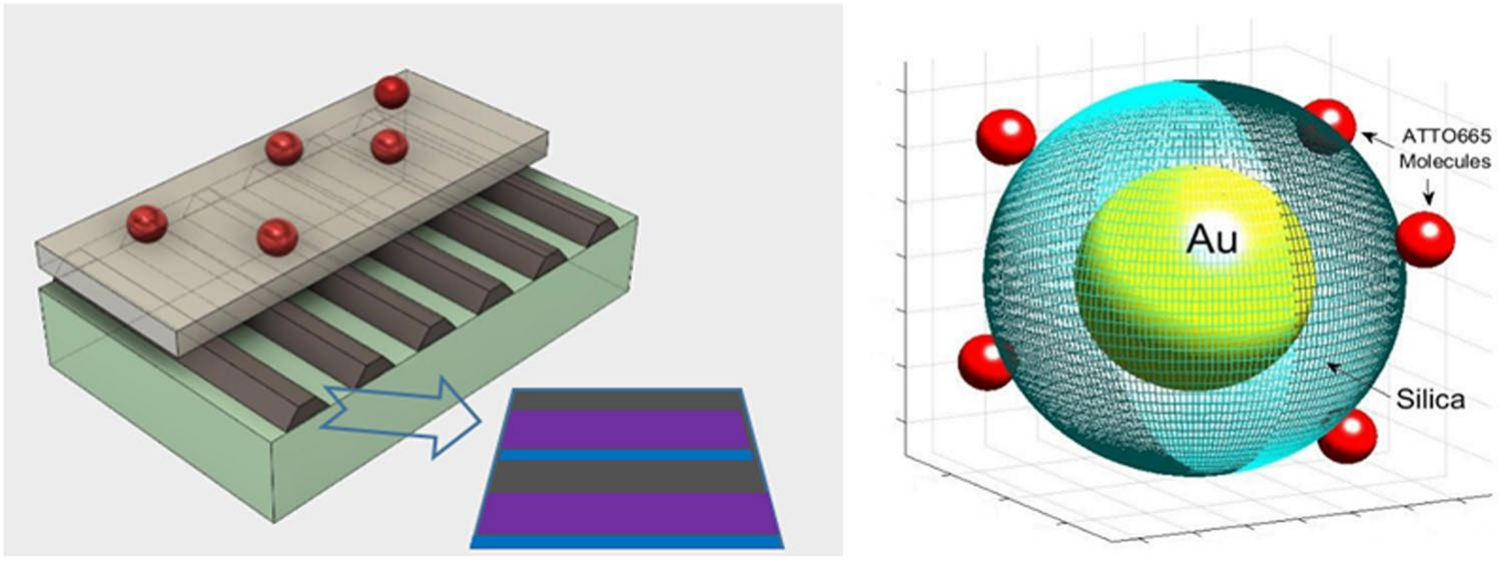
The models of the experimental and theoretical samples. Left - gratings with Rh800 molecules on top, grating strips substructure: Ti 5 nm (blue), 30 nm Ag (purple), 40 nm alumina (brown), 5 nm Ti (blue), 30 nm Ag (purple), and 10 nm alumina (brown). The periodicity of the gratings is ~310 nm. Right – Core shell nanoparticles Au/silica/ATTO655.

In this paper we report theoretical and experimental studies of bi-exponential decay of dye Rhodamine 800 (Rh800) near the metamaterial anisotropic mirror and ATTO655 in core-shell particles (Fig. 1). The superradiant and subradiant states by the identical permutational symmetry of the atom-field interaction Hamiltonian of the two-level system are distinct and exclusive. Our analytical approach shows that starting from two emitters, coupled for example via the plasmon modes, symmetry breaking occurs, making both the fast super-radiance and the slow sub-radiance observable as an intrinsic property of the system. Even if we have a group of many coupled emitters or many groups of coupled emitters with different coupling parameters, the averaging will affect the emission among the super-radiant fast transitions and in the sub-radiant slow transitions keeping these two groups distinct. Indeed, all Dicke states can be exclusively divided into two groups. One set is the so-called super-radiant states. These states have transitions from the most excited state to the ground state. The second group of states is the sub-radiant states. They have weak transitions between each other. This second group have transitions to the ground state only if they are coupled to super-radiant group of states. These sub-radiant states can trap some population of excited states since the trapped population cannot reach the ground state. Relaxation from the subradiant states in the system occurs due to radiative coupling to the vacuum modes and due to interactions with phonons in the atomic matrix and the metamaterial structure. The relaxation leads to the population of the sub-radiant states by dephasing the super-radiant Dicke states giving rise to a bi-exponential decay that is observed in the experiments and shown in the theoretical section.

The bi-exponential decay results from the cooperative atomic behavior via the relaxation processes that transfer population from the super radiative states to the non-radiative states. A set of metamaterial samples consisting of gratings of paired silver nanostrips used in our experiments is coated with Rh800 dye molecules and exhibits the expected bi-exponential behavior. The dye and metamaterial have optical resonances in the same spectral range. Note that the same layer of dyes outside the metamaterial area show a single exponential decay, which means no coupling. Thus one attributes the coupling caused by interaction via plasmon modes of metamaterial. The bi-exponential decay is demonstrated also for Au\SiO_2_\ATTO655 core-shell nanoparticles. By varying of silica spacer thickness one can vary the coupling parameter. The effect persists even when averaging over a broad range of the coupling parameter. The experimental polarization and resonance wavelength dependence of the effect is observed and discussed.

## Theory

We consider the system containing a few two-level atoms. The Hamiltonian of the system under consideration is given by

1 $$\hat{H}={\hat{H}}_{0}+{\hat{V}}_{I}+{\hat{V}}_{bath},$$

where

2 $${\hat{H}}_{0}=\hslash \sum _{j}{\omega }_{ab}|{e}_{j}\rangle \langle {e}_{j}|$$

describes the atoms (j summing over atoms), ${\hat{V}}_{I}$ describes the interaction of the atoms with external laser fields, ${\hat{V}}_{bath}$ describes the interaction with field reservoir, and takes into account the dephasing of atomic transition by phonons. The interaction picture Hamiltonian can be written, for interaction with external laser field ${E}_{L}$ as

3 $${\hat{V}}_{I}=-\,{\wp }_{ab}\sum _{j}{E}_{L}^{j}(t)({e}^{i\delta \omega t}{\sigma }_{-}^{j}+{e}^{-i\delta \omega t}{\sigma }_{+}^{j})$$

where $\delta \omega ={\omega }_{ab}-{\omega }_{L}$ is the detuning of laser frequency from the atomic transition, the interaction with baths leads to relaxation that can be described by

4 $${\hat{V}}_{bath}=\hslash \sum _{j}\sum _{k}({g}_{k}({\hat{b}}_{k}^{+}{\sigma }_{-}^{j}{e}^{i\delta {\omega }_{k}t}+{\hat{b}}_{k}{\sigma }_{+}^{j}{e}^{-i\delta {\omega }_{k}t})+\hslash \sum _{j}{{\rm{\Gamma }}}_{p}^{j}{\sigma }_{z}^{j},$$

where $\delta {\omega }_{k}={\omega }_{ab}-{\omega }_{k}$ is the frequency of vacuum modes from the atomic transition, ${\hat{b}}_{k}^{+}$ and ${\hat{b}}_{k}$ are the creation and annihilation operators for corresponding vacuum modes, ${g}_{k}=\sqrt{\frac{\wp \hslash \omega }{V}}$ is the coupling of atomic system with vacuum radiation modes, ${{\rm{\Gamma }}}_{{p}}^{{j}}$ is the perturbation of the energy of the atomic levels via the interaction with atomic environment, such as phonons, surface plasmons, etc. The interaction with the bath can be rewritten in more compact form as

5 $${\hat{V}}_{bath}=\sum _{j}({\wp }_{ab}({\hat{E}}_{j}^{+}(t){\sigma }_{-}^{j}+{\sigma }_{+}^{j}{\hat{E}}_{j}(t))+\hslash {{\rm{\Gamma }}}_{p}^{j}(t){\sigma }_{z}^{j}),$$

where we introduce the vacuum fields operators ${\widehat{\xi }}_{j}^{+}(t)=\sum _{k}{\xi }_{0}{\hat{b}}_{k}^{+}{e}^{i\delta {\omega }_{k}t}$ and ${\widehat{\xi }}_{j}(t)=\sum _{k}{\xi }_{0}{\hat{b}}_{k}{e}^{-i\delta {\omega }_{k}t}$, ${\xi }_{0}=\sqrt{\frac{2\hslash \omega }{{\varepsilon }_{0}V}}$ is the electric field per photon, and $V$ is the quantization volume.

The master equation taking into account the relaxation terms can be obtained by using Born-Markov approximation

6 $$\dot{\rho }={(\frac{1}{i\hslash })}^{2}{\int }_{0}^{t}dt^{\prime} {{\rm{Tr}}}_{R}([{V}_{I}(t),[{V}_{I}(t^{\prime} )\rho \oplus {\rho }_{R}]])$$

The averages for fields are $\langle {{\rm{\Gamma }}}_{j}(t)\rangle =0$, $\langle {\widehat{\xi }}_{l}^{+}(t^{\prime} )\rangle =0$, $\langle {\widehat{\xi }}_{l}(t^{\prime} )\rangle =0$, $\langle {{\rm{\Gamma }}}_{j}(t){\widehat{\xi }}_{l}^{+}(t^{\prime} )\rangle =0$, $\langle {{\rm{\Gamma }}}_{j}(t){\widehat{\xi }}_{l}(t^{\prime} )\rangle =0$. The vacuum reservoir has no phase correlations, i.e. ${\langle {\widehat{\xi }}_{j}(t){\widehat{\xi }}_{l}(t^{\prime} )\rangle }_{R}=0$, ${\langle {\widehat{\xi }}_{j}^{+}(t){\widehat{\xi }}_{l}^{+}(t^{\prime} )\rangle }_{R}=0$.

The relaxation due to coupling to vacuum fields depends on correlations of vacuum fields for different atoms, i.e.

7 $${\wp }_{ab}^{2}{\langle {\widehat{\xi }}_{j}(t){\widehat{\xi }}_{l}^{+}(t^{\prime} )\rangle }_{R}=\hslash \gamma {f}_{jl}\delta (t-t^{\prime} ).$$

In our case, the atoms are located within the wavelength of optical radiation, so we assume that ${f}_{jl}=1$. For dephasing rates, we assume that

8 $$\langle {{\rm{\Gamma }}}_{j}(t){{\rm{\Gamma }}}_{l}(t^{\prime} )\rangle ={{\rm{\Gamma }}}_{p}{f}_{jl}^{p}\delta (t-t^{\prime} ),$$

and we consider local as well as nonlocal contribution to relaxation.

The Master equation for the density matrix for the atomic system is given by

9 $$\dot{\rho }=\gamma (2{\sigma }_{-}\rho {\sigma }_{+}-{\sigma }_{+}{\sigma }_{-}\rho -\rho {\sigma }_{+}{\sigma }_{-})-{{\rm{\Gamma }}}_{p}[{\sigma }_{z},[{\sigma }_{z},\rho ]]-\sum _{j}{\Gamma }_{pj}[{\sigma }_{z}^{j},[{\sigma }_{z}^{j},\rho ]],$$

where $\rho$ is the density matrix, ${\Gamma }_{p}$ and ${\Gamma }_{pj}$ are the nonlocal and local dephasing rates.

The presence of the metamaterial structure influences strongly the interaction between atoms as well as interaction with “vacuum” fields modifying the spontaneous relaxation and dephasing rates are modified. The Purcell factor, as shown in^3^, is given by

10 $${F}_{p}^{z}=\frac{3\pi }{8{k}^{3}}{({k}_{||})}^{2}\delta {k}_{||}=\frac{3\pi }{8{k}^{3}{d}_{1}^{3}}{\mathrm{ln}}^{2}|\xi |{\xi }^{\frac{{d}_{2}-{d}_{1}}{2{d}_{1}}},$$

where $\xi =-\,\frac{{\varepsilon }_{1}{\varepsilon }_{2}}{{({\varepsilon }_{1}+{\varepsilon }_{2})}^{2}}$, and ${\varepsilon }_{1}$, ${\varepsilon }_{2}$ are the permittivities of metal and dielectric. The equation (10) is valid at the plasmon resonance appearing if the real part of the denominator ${\varepsilon }_{1}+{\varepsilon }_{2}$ aproaches zero. The excitation of the surface plasmon polaritons has polarization dependence. Indeed, for a multilayer structure, the effective dielectric constant depends on polarization

11 $${\varepsilon }_{eff}={\varepsilon }_{\perp ,||}\approx {(\frac{{\varepsilon }_{1}^{\alpha }{d}_{1}+{\varepsilon }_{2}^{\alpha }{d}_{2}}{{d}_{1}+{d}_{2}})}^{\frac{1}{\alpha }}$$

where ${d}_{1,2}$ are the thickness of layers in the structure, and ${\varepsilon }_{1,2}$ are the dielectric constants of the layers, and parameter $\alpha =1$ for the parallel orientation of the polarization and $\alpha =-\,1$ for the perpendicular orientation. The maximums of the factors are the same order of magnitude (see ref.^3^), ${F}_{p}^{z} \sim {F}_{p}^{x}$ for $|\xi |\gg 1$, and by changing the parameters, for example the thickness ${d}_{1}$ and ${d}_{2}$ of the metamaterial structure, one can control the coupling to the structure.

In the case of metamagnetic gratings the magnetic resonance wavelength ${\lambda }_{m}$ depends on the geometric parameters of the paired-strip structures: $w$, $t$ are the width and thickness of the metal strips, and $d,\,{n}_{d}$ are the thickness and refractive index of the dielectric spacer between two metal strips. Following the cavity model approach discussed in ref.^4^, for the range of experimental parameters ${\lambda }_{m} > 2{n}_{d}d$, the resonant wavelengths ${\lambda }_{m}$ are well described by^5^

12 $${\lambda }_{m}={\lambda }_{p}\sqrt{4+{(\frac{{n}_{d}^{2}w}{\pi t})}^{2}+(\frac{2{n}_{d}^{2}{w}^{2}}{{\pi }^{2}td})},$$

where ${\lambda }_{p}$ is the plasma wavelength, which is approximately 136 nm for Ag and Au. This relation we used to design gratings with magnetic resonance varying in the range of the luminescence band of dye Rhodamine 800.

In the simulation, we use the spontaneous rate in vacuum for dye molecules to be $\gamma \approx {10}^{8}$ s^−1^. To describe the coupling factor between dye molecules and the metamaterial structure, we introduce the factor $G$ such as $G\approx {{F}_{p}}^{x} \sim {F}_{p}^{z}$. Since the Purcell factor depends on the emission frequency shift with respect to plasmon resonance the coupling factor $G$ will be different for gratings with different ${\lambda }_{m}$. The spontaneous rate is modified as $\gamma ={\gamma }_{vac}(1+G)$ as well as the dephasing rates ${{\rm{\Gamma }}}_{pj}={\beta }_{i}G\approx \beta G$. The rate of inhomogeneous broadening was used to be ${{\rm{\Gamma }}}_{p}\approx {10}^{9}$ s^−1^, generally the inhomogeneous broadening has broadening with long wavelength phonons ${{\rm{\Gamma }}}_{p}$ and short wavelength phonons as ${{\rm{\Gamma }}}_{pj}$. We assume that the radiation modes are overlapped, so ${f}_{jl}\approx 1$ (in general, the relaxation part can include also surface plasmon radiation modes with short wavelength describing by factors ${f}_{jl}^{p}$).

**Figure 2:**
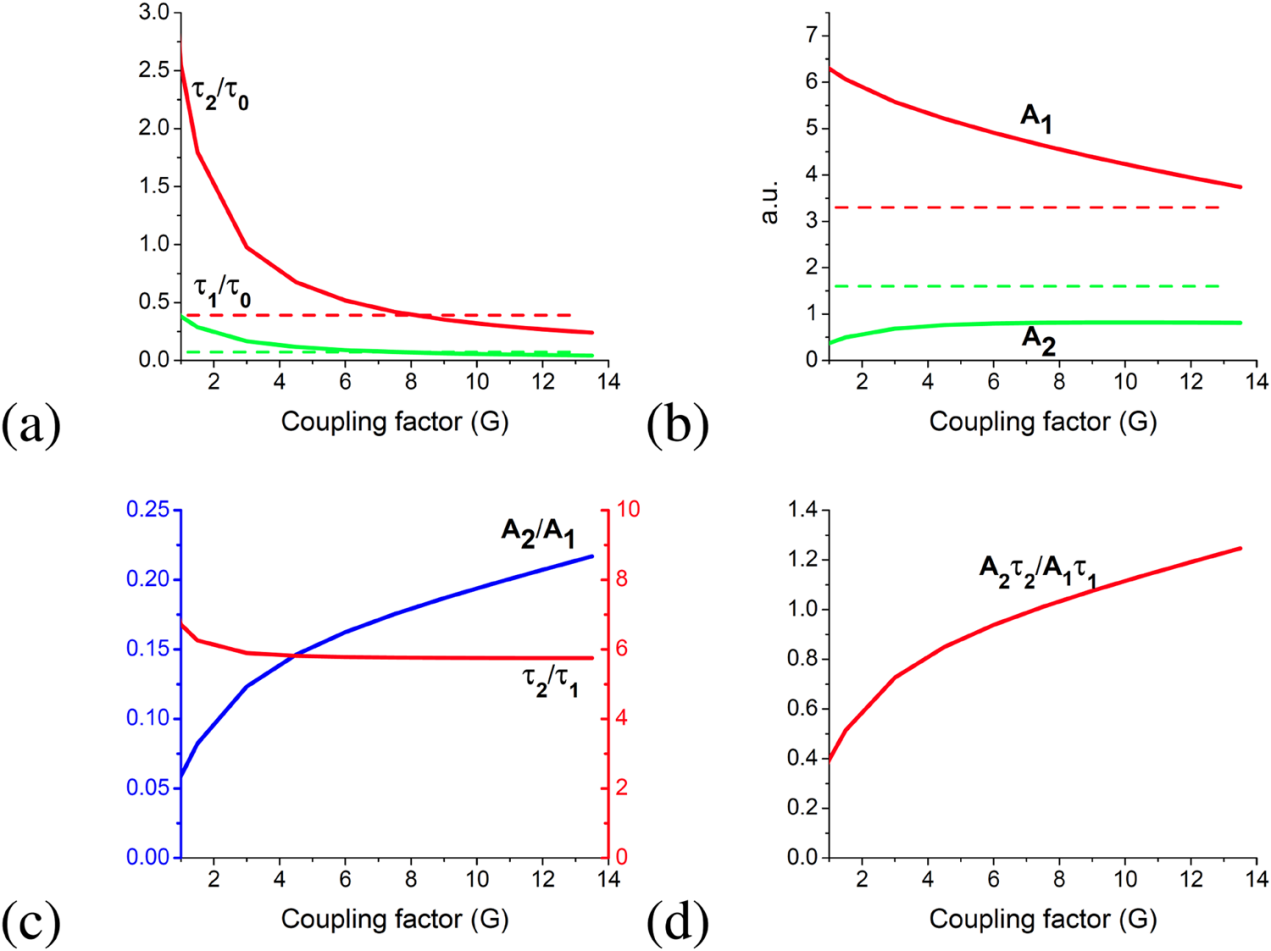
Bi-exponential fitting of theoretical results versus the effective coupling to the plasmonic structure. (**a**) The superradiance and subradience decay times ${\tau }_{1}$ and ${\tau }_{2}$ normalized by the decay time without metamaterial ${\tau }_{0}$. Dashed lines show average of ${A}_{1}{e}^{-t/{\tau }_{1}}+{A}_{2}{e}^{-t/{\tau }_{2}}$ over 0 < G < 14 (**b**) the amplitudes at the exponents ${A}_{1}$ and ${A}_{2}$, dashed lines are average. (**c**) A ratio of the amplitudes, and a ratio of the decay times, (**d**) a ratio of the total number of photons (a product of the amplitude and life time).

Simulations have been performed for the case of two and three atoms. The results for three atoms are presented in Fig. 2. We assume that three atoms are coupled to the same vacuum modes. The phase decay has two parts, one is coupling to the same bath (it describes the homogeneous broadening of coherence), and another part is coming from the coupling to the metamatrial structure (near fields, surface plasmons, etc.). Performing the simulations, we vary the coupling parameter to the metamaterial structure. Without coulping to the metamaterial structure, the only single-exponential decay can be observed.

Once we have the coupling to the metamaterial structure, the results show the bi-exponential decays, ${A}_{1}{e}^{-t/{\tau }_{1}}+{A}_{2}{e}^{-t/{\tau }_{2}}$ with the parameters shown in Fig. 2. The results qualitatively agree with the experimental results presented below. We can see that an isolated atom has only one relaxation rate. When the system contains two or three atoms, the theory predicts the bi-exponential decay near the metamaterial structure that depends on the parameter of coupling to the structure. The decays related to the cooperative superradiant behavior of the atoms interacting with the same vacuum fields and related to the populations of the nondecaying states by local depahsing.

The experiments have been performed with subwavelength gratings or core-shell particles. The gratings have a vertical substructure in each strip that support a plasmon resonance, which can be tuned by changing the strips vertical geometry. The strips were designed to have a plasmon resonance of the assymetrical mode in the spectral range covering the dye absorption/emision maxima. By changing the resonance wavelength and consequently spectral difference relative to the emission maximum, one can realize the different coupling parameters (decreasing if the spectral difference increases). The incident light with polarization parallel to the strips sees the sample as diluted metal mirror with dye film. Thus in this case the coupling is less than for the perpendicular polarization and similar between all four samples. The core-shell nanoparticles have a broad variation in the dielectric spacer thickness between Au core and ATTO655 dye layer in the experiments, thus providing a case of average signal over broad range of coupling parameters. The theoretical averaging was done over the coupling parameter ranging from 0 to 14.

The results of the theory shown in Fig. 2 highlight several key features. As the coupling parameter G grows from zero, the super-radiance time reduces from ${\tau }_{0}$ to ${\tau }_{1}(G=4)\approx 0.17{\tau }_{0}$. The slow exponent time gets down to ${\tau }_{2}(G=4)\approx {\tau }_{0}$(Fig. 1a). Both, slow and fast life times are less than ${\tau }_{0}$ at G > 4. The ratio of slow to fast times saturates at G > 1. The ${\tau }_{2}/{\tau }_{1}$ ratio increases with G going to zero but ${A}_{2}$ becomes negligible. The slow exponent’s amplitude, ${A}_{2}$, is growing from zero, and the growth gets saturated at G > 1. Note also, that the number of photons in the sub-radiance pulse and the super-radiance pulse are of the same order at high coupling parameter, specifically the ratio of number of photons increases from 0.8 to 1.2 as G increases from 4 to 12. Coupling of the dye molecules via the plasmonic surface results in collective modes, superradiant and subradiant. The coupling speeds up the emission of the superradinat mode. The subradiant modes have no ground states, consequently can be excited only through the coupling mechanism, sharing population from the superradiant modes. That is the reason why subradiant decay time is always longer and the amplitude is always less. Due to such an intrinsic link between two regimes the collective modes are always distinct and shown up in the form of fast and slow exponential decay. Implying that there are many groups of emitters coupled with different coupling parameter G one should average the emission decay over G. Figure 2(a) shows two dashed lines obtained by the integration ${A}_{1}{e}^{-t/{\tau }_{1}}+{A}_{2}{e}^{-t/{\tau }_{2}}$ over 0 < G < 14. Since all of the groups have a high intrinsic ratio between the slow and fast times of the bi-exponential decay, such an averaging will preserve bi-exponential behavior of the decay with almost the same ratio ${\tau }_{2}/{\tau }_{1}\approx 5.5$. Note, that those lines are at the middle of the integration range and in good correspondence with ${\tau }_{1}$ and ${\tau }_{2}$ without averaging. That is a strong evidence of the intrinsic exclusive properties of the coupling in each group of the coupled emitters. What is counterintuitive though is that the amplitudes of two exponents, ${A}_{1}$ and ${A}_{2}$ for the average decay do not even cross the curves at any coupling parameter in the range (Fig. 2b). However similar result is obtained in the experiments, i.e. the ${A}_{2}/{A}_{1}$ ratio is close to 0.5 in the case of core-shell Au/SiO_2_/ATTO655 nanoparticles.

## Experimental Results: Rhodamine 800 on Gratings

**Figure 3:**
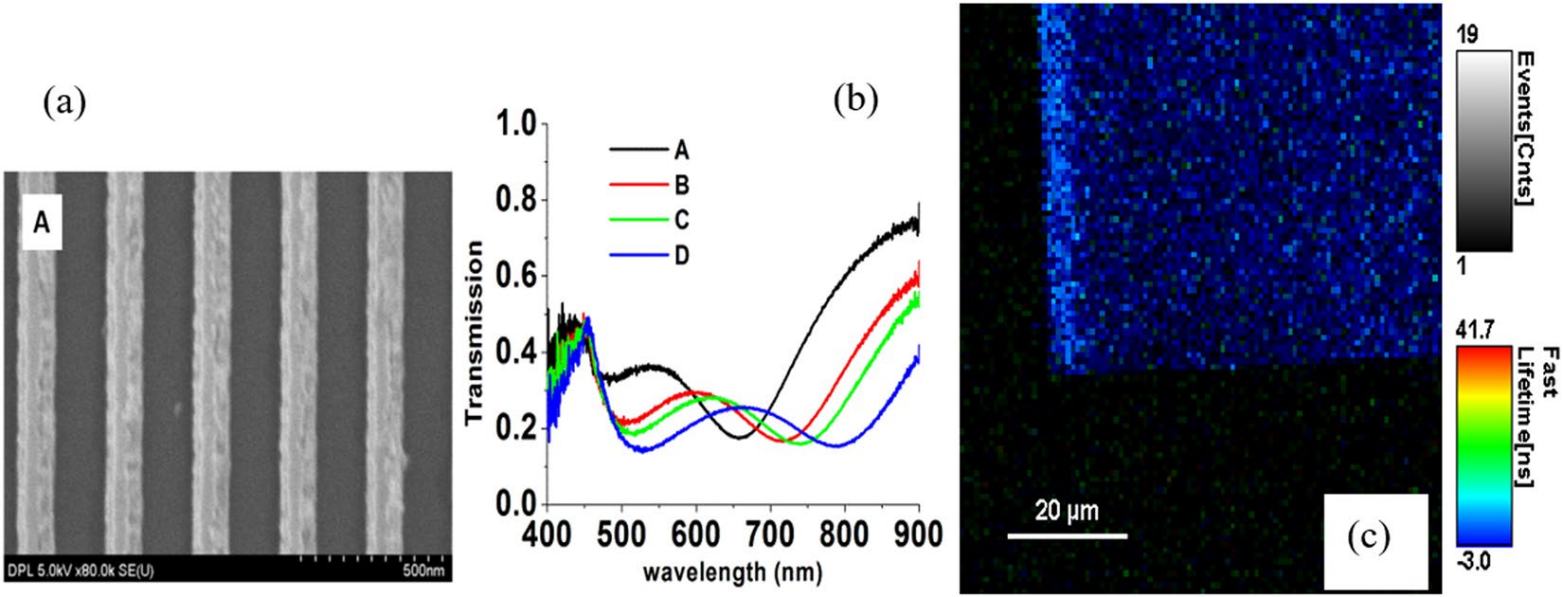
(**a**) SEM image of one of the grating samples (A). Gratings strips substructure: Ti 5 nm, 30 nm Ag, 40 nm alumina, 5 nm Ti, 30 nm Ag, and 10 nm alumina. The periodicity of the gratings was ~310 nm. The bottom width of the trapezoidal gratings are 138.2 nm (±6.5 nm), 156.6 nm (±7.1 nm), 154.4 nm (±8.0 nm), and 168.9 nm (±7.0 nm) for A, B, C, and D, respectively. The top width of the trapezoidal gratings are 52.7 nm (±4.5 nm), 65.4 nm (±7.5 nm), 69.8 nm (±8.1 nm), and 84.0 nm (±7.6 nm) for A, B, C, and D, respectively. (**b**) Transmission spectra of the A, B, C, D gratings at TM polarization. (**c**) FLIM image of the grating corner. Brightness bar shows that PL of Rh800 is enhanced at the grating location. Color bar indicates life time measured in a fast mode using the single exponent fitting.

The paired Ag nanowire gratings were fabricated by e-beam lithography as described in the section Methods. The procedure includes deposition of titanium (Ti) adhesion layer, silver (Ag), alumina (Al_2_O_3_), Ag, and Al_2_O_3_ as a protective layer with an e-beam evaporator at a rate of 0.5 Å/s for Ti and Ag and 1.0 Å/s for Al_2_O_3_. The structure of one of four samples is shown with a field emission scanning electron microscopy (FE-SEM) image in Fig. 3a. Transmission spectra in Fig. 3b are indicative of two minima in accord with two resonances, symmetrical and asymmetrical modes^5,6^. Reflection spectra with retrieved phase spectra can be seen in the supplementary information.

**Figure 4:**
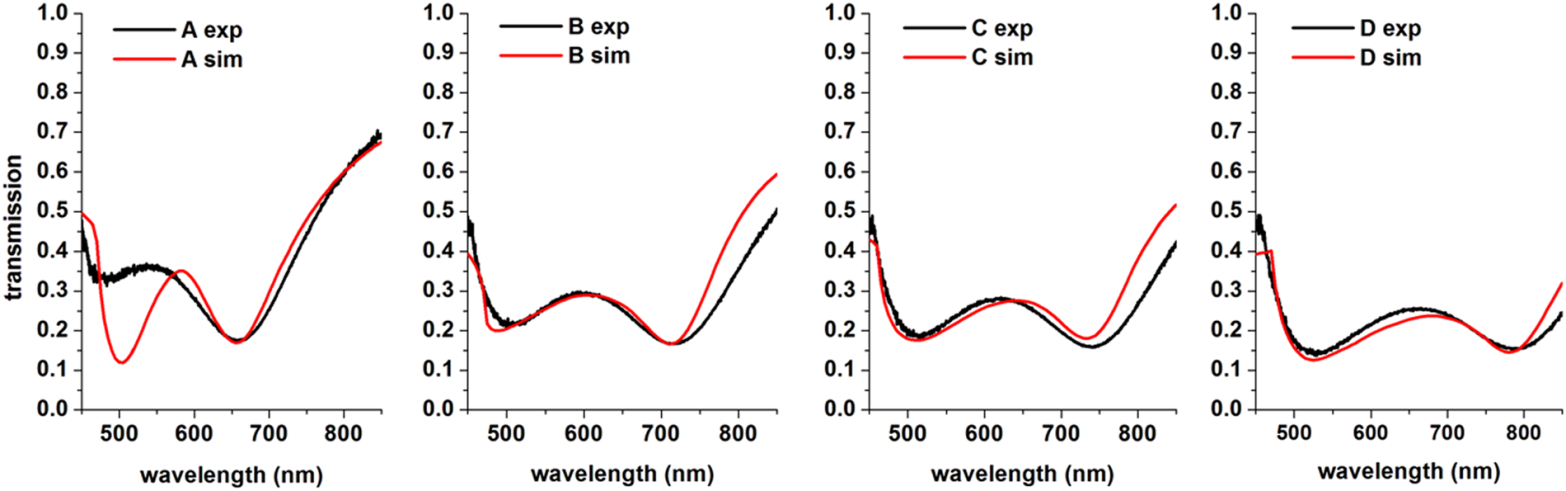
Matching the experimental spectra with SHA simulations. The matching for Die A is insufficient at shorter wavelengths since the quality of the written area was not as good as the others.

Fluorescent lifetime image microscopy (FLIM) was done with a pulse diode laser of 640 nm wavelength on paired Ag nanowire grating samples that had been spin coated with a spacer layer (30 nm) then a 100 μM Rhodamine 800 dye in SU-8 layer (22 nm) (see Methods for details). In Fig. 3b, the fabrication of uniform gratings produces electric resonance around 500 to 525 nm and magnetic resonance spanning 650 nm to 800 nm. The magnetic resonance of the gratings are excellent candidates to overlap with the Rh800 dye absorption maximum at 690 nm and emission maximum at 720 nm as presented in the Supplementary information. As the width of the gratings is increased the magnetic and electric resonances red shifts to higher wavelength with TM illumination; the gratings behave as a dilute metal for TE polarization^5–8^. In Fig. 4, the experimental spectra were matched using spatial harmonic analysis (SHA)^7^.

The photoluminescence decay (see Methos) were fitted with the exponential reconvolution using measured instrumental reference function. All the data were fitted with a single-, bi-, or triple- exponential function, then the best fit is selected. It is proven that the time dependence of photoluminescence decay for Rh800 in the vicinity of the gratings is bi-exponential, ${A}_{1}{e}^{-t/{\tau }_{1}}+{A}_{2}{e}^{-t/{\tau }_{2}}$, with a fast super-radiant mode and a slow subradiant emission. Fitting with three exponential terms gives the amplitude of third term negligible relative to the other two terms. The best fitting is characterized by χ^2^, which should be close to unity for the best fit. If we would try to fit it with a single exponent, χ^2^ would be 1.4, which indicates that this option is not acceptable. The results for four dies A, B, C, D are combined in the Table 1.

**Table 1:** Experimental data summary for grating with Rh800 in SU8 layer. *A*_1_*, A*_2_*, τ*_1_*, τ*_2_ are amplitudes and life times of two exponents, *τ*_0_ = 2.4 ns.

	A_para_	B_para_	C_para_	D_para_	A_ortho_	B_ortho_	C_ortho_	D_ortho_
A_2_/A_1_	0.1	0.1	0.11	0.12	0.38	0.42	0.58	0.68
τ_2_/τ_1_	4.66	4.73	4.71	4.9	4.1	3.6	3	3
τ_2_ ns	1.4 ± 0.025	1.42 ± 0.025	1.46 ± 0.015	1.57 ± 0.02	1.8 ± 0.02	1.8 ± 0.02	1.8 ± 0.024	1.8 ± 0.012
τ_1_ ns	0.3 ± 0.005	0.3 ± 0.006	0.31 ± 0.003	0.3 ± 0.005	0.44 ± 0.01	0.5 ± 0.015	0.6 ± 0.014	0.6 ± 0.012
τ_2_/τ_0_	0.58	0.58	0.6	0.61	0.75	0.75	0.75	0.75
τ_1_/τ_0_	0.125	0.125	0.13	0.133	0.18	0.21	0.25	0.25
G	G_A_	G_B_	G_C_	G_D_	G_Ares_	G_Bres_	G_Cres_	G_Dres_

The data are shown for dies A, B, C, D at parallel polarization of the incident light (…)_para_, and polarization orthogonal to the grating strips (…)_ortho_. Bottom row sets notations for corresponding coupling parameters.

## Experimental Results: ATTO655 Dye in the Shell of Au/SiO_2_ Core

Synthesis of Au\SiO_2_ core-shell is done under vigorous stirring of 1 mL of Au 20 nm colloids concentration at 7 × 10^11^ particles/mL in 250 mL of 200 proof ethanol solution^8^ as described in Methods. The fluorescent-coating of Au\SiO_2_ core-shell is followed by doping method with 6.2μM ATTO655 ethanol solution. The core-shells were once again separated re-dispersed in 10% SU8 and spin coated on a thin glass substrate (see Methods).

**Figure 5:**
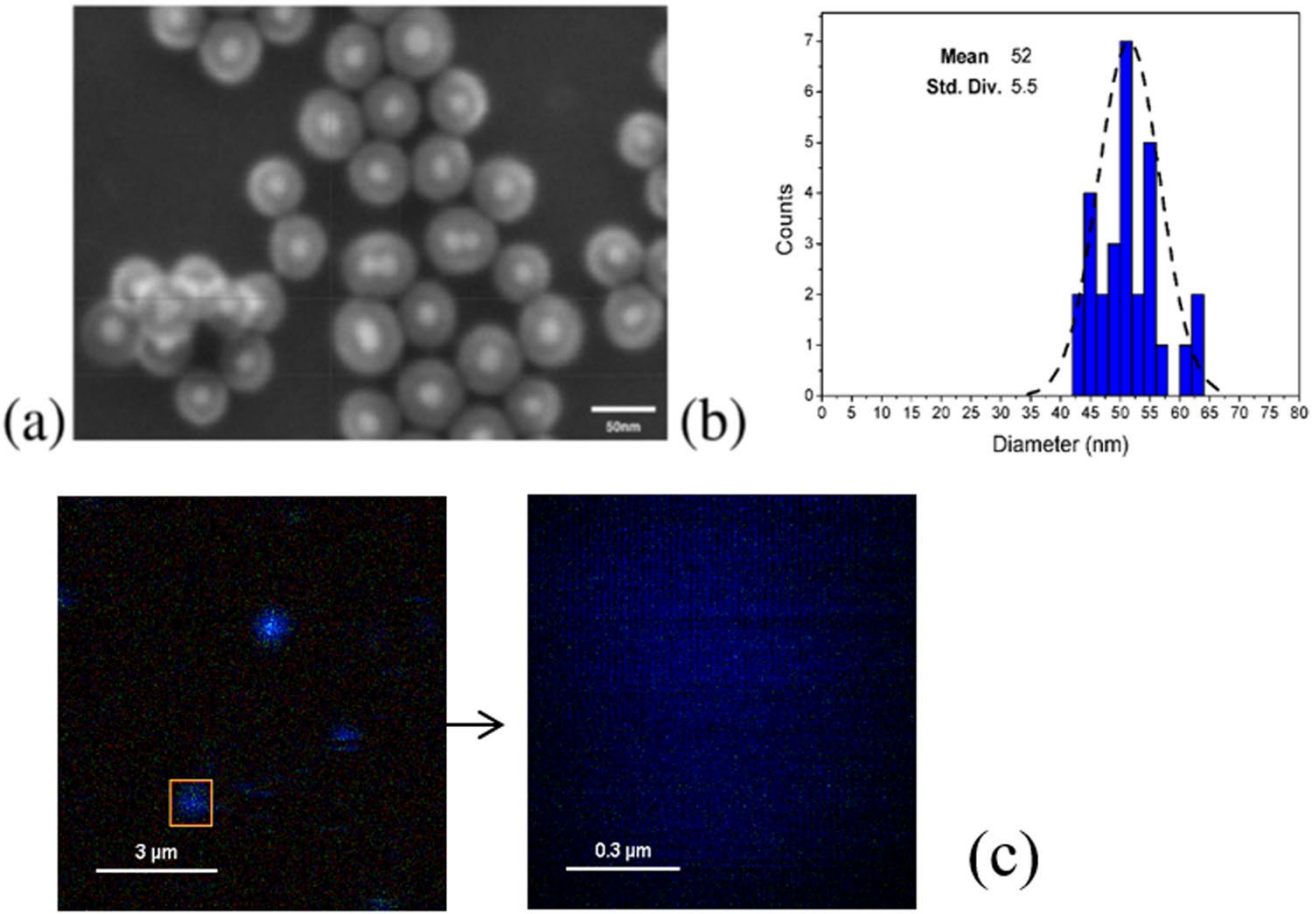
(**a**) FE SEM image made on Si substrate scale bar 50 nm, (**b**) particle distribution, mean 52 nm. (**c**) FLIM images of the core-shell particles deposited on a substrate. The bright area were selected for the life time measurements. These bright area can contain a group of core-shells.

The broad size distribution in Fig. 5b tells that the sample contains groups of emitter with quite broad range of the coupling parameters. The particle distribution over a substrate shows (see SI) that the signal may contain contribution from different groups of core-shell nanoparticles.

The ATTO655 life time measured in the SU8 epoxy film deposited on a substrate and dried is varied from 1.8 to 3.2 ns depending on the film preparation method and collection area location (see Table 2a). The fluorescence life time microscopy (FLIM) images with two different scales in.

**Table 2:** (a) Lifetime measurements ATTO655 in 10% SU8. Sample1: ATTO655 313 μM droplet; Sample 2, 3: ATTO655-5nM in 10% SU8 Matrix, 2000 rpm; Sample 4: ATTO655 5 nM in 10% SU8 Matrix, 2000 rpm. Collection area about 0.4 μm^2^. Single exponential decay. λ = 640 nm, NA=1.2. (b) Lifetime measurements for ATTO655 coated on Au-SiO_2_ in 10% SU8 spin coated on a glass substrate. Samples 5, 6, 7, 8, 9: Au-SiO_2_-ATTO655 in 10% SU8 Matrix, 2000 rpm. Bi-exponential decay. λ = 640 nm, NA=1.2.

Sample	I_1_ Kcnts	A_1_ Kcnts	τ_1_ ns	χ^2^			
1 (droplet)	499	2.5	3.2 ± 0.003	1.062			
2 (spin)	90	0.66	2.2 ± 0.007	1.035			
3 (spin)	78	0.568	2.2 ± 0.006	1.041			
4 (spin)	11	0.094	1.8 ± 0.033	1.083			
**Sample**	**I**_**1**_ **Kcnts**	**I**_**2**_ **Kcnts I**_**2**_ **/I**_**1**_	**A**_**1**_ **Kcnts**	**A**_**2**_ **Kcnts A**_**2**_ **/A**_**1**_	**τ**_**1**_ **, ns**	**τ**_**2**_ **, ns τ**_**2**_ **/τ**_**1**_	**χ** ^**2**^
5	13	7.7 0.6	0.43	0.048 0.11	0.46 ± 0.005	2.6 ± 0.1 5.65	1.019
6	38	69 1.8	0.51	0.340 0.66	1.2 ± 0.06	3.24 ± 0.05 2.7	1.038
7	133	91 0.68	1.6	0.4 0.25	1.34 ± 0.02	3.6 ± 0.04 2.7	0.961
8	96	110 1.1	1.56	0.56 0.36	1 ± 0.02	3.1 ± 0.02 3.1	0.988
9	19	8 0.42	0.7	0.06 0.086	0.44 ± 0.004	2.5 ± 0.1 5.68	1.010

Figure 5(c) show randomly distributed Au\SiO_2_\ATTO655 core-shell particles on a substrate. The life time measurements were performed using the higher magnification images. In all theses cases the single exponential decay occurs for the dye/SU8 film without core shell structure. The decay behavior for ATTO655 on the Au\SiO_2_ core-shell becomes bi-exponential ${A}_{1}{e}^{-t/{\tau }_{1}}+{A}_{2}{e}^{-t/{\tau }_{2}}$ as Table 2b shows using notation *I*_1_ and *I*_2_ for the total number of photons corresponding to the first and second exponents integrated over time. Note, that there are two types of samples in the Table 2(b). If we look at the data for ${A}_{2}/{A}_{1}$ and ${\tau }_{2}/{\tau }_{1}$ ratio for the samples 5 and 9 we have all the parameters in the range predicted by theory in Fig. 2 for different coupling G. For samples 6, 7, 8 all the parameters are out of the range for particular coupling parameters, but in the range for the average data.

## Discussion

**Figure 6:**
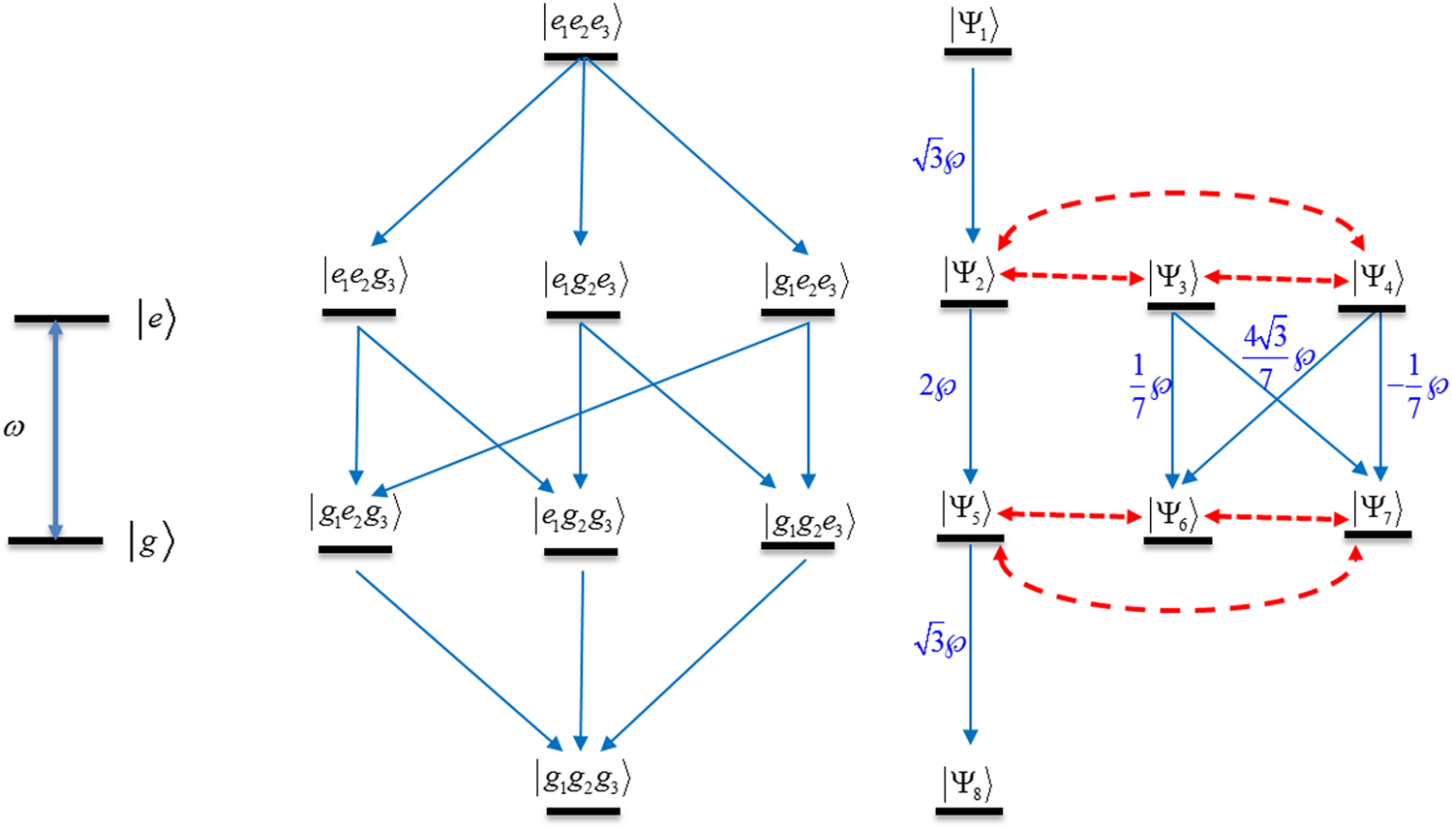
The scheme of energy level for the case of a single two -level atom (*Left*); the scheme of energy level for the case of three two-level atoms: (*Middle*) bare state basis and (*Right*) the Dicke state basis. The dipole-allowed transitions are shown by blue lines. The mixing between Dicke states are shown by the dashed red lines.

Thus our experiments and theory show the intrinsic properties of coupled atoms. It is evident from the following analysis in the case of three atoms. The theoretical approach employs the full set of coupled system. We use the basis of the so-called Dicke states. For example, the structure of three atomic system levels is shown in Fig. 6. It is clear seen that all Dicke states can be divided into two groups. One set is the so-called super-radiant states. These states have transitions between each other from the most excited state to the ground state. The second group of states is the sub-radiant states. They have weak transitions between each other. This second group have no ground state if they are decoupled from super-radiant states.

The Dicke states shown in Fig. 6 are given by

13 $$\begin{array}{c}|{{\rm{\Psi }}}_{1}\rangle =|eee\rangle \\ |{{\rm{\Psi }}}_{2}\rangle =\frac{1}{\sqrt{3}}(|eeg\rangle +|ege\rangle +|gee\rangle )\\ |{{\rm{\Psi }}}_{3}\rangle =\frac{1}{\sqrt{14}}(|eeg\rangle +2|ege\rangle -3|gee\rangle )\\ |{{\rm{\Psi }}}_{4}\rangle =\frac{1}{\sqrt{42}}(5|eeg\rangle -4|ege\rangle -1|gee\rangle )\end{array}$$

14 $$\begin{array}{c}|{{\rm{\Psi }}}_{5}\rangle =\frac{1}{\sqrt{3}}(|egg\rangle +|geg\rangle +|gge\rangle )\\ |{{\rm{\Psi }}}_{6}\rangle =\frac{1}{\sqrt{14}}(|egg\rangle +2|geg\rangle -3|gge\rangle )\\ |{{\rm{\Psi }}}_{7}\rangle =\frac{1}{\sqrt{42}}(5|egg\rangle -4|geg\rangle -1|gge\rangle )\\ |{{\rm{\Psi }}}_{8}\rangle =|ggg\rangle \end{array}$$

It is clearly seen that the sub-radiant states can trap some population and the trapped population cannot reach the ground state. The relaxation in the system occurs due to radiative coupling to the vacuum modes and due to the interaction with the phonon in the atomic matrix and the metamaterial structure. The relaxation leads to the population of the sub-radiant states by dephasing the super-radiant Dicke states giving rise to the bi-exponential decay that was observed in the experiments as shown in Fig. 6 by dashed red lines. Thus, the bi-exponential decay results from the cooperative atomic behavior via the relaxation processes that transfer population from the super radiative states to the non-radiative states.

In our theoretical approach, we have considered mechanism of coupling between bright, decaying, states and dark, decoupled, states. The one includes the dipole-dipole interaction between molecules which leads to the different relaxation times when the molecules are distributed not homogeneously. The second one is related to the phase relaxation that can involve interaction with phonons, surface plasmons, etc. leading to the relaxation of coherences, but not populations of the molecular levels. Due to dephasing, the Dicke states and the dark states turned out to be interacting with each other leading to the modification of the relaxation rates. The relaxation of Dicke states becomes slightly slower, but this interaction speeds up significantly the decay of dark states (see Fig. 2 showing the change of the ${\tau }_{1}\,{\rm{and}}\,{\tau }_{2}$ versus coupling) resulting in bi-exponential decays of population of molecular states.

The different molecules are located in different environment of the metamaterial grating, and they have different coupling to the radiation modes of the grating as well as they have different coupling with local and non-local phonons and plasmons of the metamaterial. Experimentally the laser radiation interacts with many molecules and detection system is also integrating the signal from many molecules. Some averaging procedure should be applied to determine the signal. Experimentally, we have some distribution of the coupling constant that should be taken into account in the theory as well. It is not obvious a-priori if the bi-exponential behavior is going to survive this averaging procedure. The averaging over full range of the coupling constant with rectangular distribution proves the intrinsic nature of the bi-exponential decay in the coupled system. The results shown in Fig. 2(a) with dashed lines prove that after the averaging of groups of coupled emitters over coupling parameter G from 0 to 14, times ${\tau }_{1}\,{\rm{and}}\,{\tau }_{2}$ are practically coincide with ones that the local coupling strength equal to the average coupling. The experimental results confirm this observation since the bi-exponential behavior does not disappear even in the system with a broad distribution of the coupling geometry (see Table 2b for core-shell emitters).

The subradiant modes have no ground states, consequently can be excited only through the coupling mechanism, sharing population from the superradiant modes. That is the reason why subradiant decay time is always longer and the amplitude is always less. Due to such an intrinsic link between two populations the collective modes are always distinct from normal modes and shown up with signature fast and slow exponential emission decay lifetimes. If we consider that there are many groups of emitters coupled with different coupling parameter, G, in a real system, one should average the emission decay over G. Figure 2(a) shows two dashed lines obtained by the integration ${A}_{1}{e}^{-t/{\tau }_{1}}+{A}_{2}{e}^{-t/{\tau }_{2}}$ over 0 < G < 14. Since all of the groups have a high intrinsic ratio between the slow and fast times of the bi-exponential decay, such averaging will reveal the bi-exponential behavior of the decay with a common ratio ${\tau }_{2}/{\tau }_{1}\approx 5.5$. Note, that the dotted average lines are at the middle of the integration range and in good correspondence with ${\tau }_{1}$ and ${\tau }_{2}$ without averaging. That is a strong evidence of the intrinsic exclusive properties of the coupling in each group of the coupled emitters. What is counterintuitive though is that the amplitudes of two exponents, *A*_1_ and *A*_2_ for the average decay do not even cross the curves at any coupling parameter in the range (Fig. 2b). However similar result is obtained in the experiments, i.e. the *A*_2_*/A*_1_ ratio is close to 0.5 in the case of core-shell Au/SiO_2_/ATTO655 nanoparticles.

It is known that, if a fluorescent molecule is placed in the vicinity of a mirror (gratings), the angular distribution and decay time of the emission are affected and dependent on the molecule-mirror separation^9–12^. This is caused by changes in the photonic density of states due to interference of the emitted wave that is reflected by the mirror and the non-reflected wave.

In the case of our samples the distance to the dye layer is the same for all gratings, but variations in the photonic density of states occur due to different phase shift of the reflected waves for different samples and different polarization of both excitation and emission. This results in changes of the coupling parameter G introduced in the theory. Thus the bi-exponential decay, which is evident in measured decay curves and following up fitting, will show difference in the amplitude ratio and time ratio extracted from bi-exponential fitting. The polarization dependences of the biexponential coefficients and lifetimes shown in Table 1 is due to the polarization dependence of the gratings’ response (see SI for the amplitude and phase spectra for two polarizations). Orientation of molecule dipole moments is random, but polarization of the excitation source makes preferable orientation of the emitting dipoles. As we know the transition dipole moment for excitation and dipole moment for emission have some fixed angle for each molecule. That is why the polarization dependence for both absorption and emission occur, meaning that it depends on the incident light polarization. This dependence is a cosine type. A specific molecule can be part of different collective groups, providing averaging of the coupling parameters. The effect of the mirror is greatest when resonance is present, having polarization orthogonal to the gratings.

What can be concluded from the obtained parameters is in qualitative agreement with theoretical results in Fig. 2. Indeed, the core-shell particles represent a broad distribution of in the particles size, shape, and aggregates. Thus the samples present an example of groups of particles with different coupling parameters and demonstrate bi-exponential decay. The pure dye films prepared with drop and dry method shows different life time with respect to the spin coated dye in epoxy films. The environment changes the quenching and consequently life time keeping a single exponential decay. The core-shell in epoxy films show always bi-exponential decay despite the broad distribution in the coupling geometry. The gratings demonstrate bi-exponential decay for the emitters places at the surface. All the gratings for parallel polarizations act as a diluted metal mirror and show very similar reflection spectra for amplitudes and phases. So the coupling parameters for dyes near these gratings at parallel polarization are approximately equal G_A_ ≈ G_B_ ≈ G_C_ ≈ G_D_. The A_2_/A_1_ and τ_2_/τ_1_ in this case are also approximately equal to each other. The orthogonal polarization makes the resonance of a grating response gradually shifted. The A_2_/A_1_ and τ_2_/τ_1_ data indicates that G_A_ < G_B_ < G_C_ < G_D_. The experimental data shows a typical ratio τ_2_/τ_1_ at the level of 3, which can be due to the average signal over different collective groups. The A_2_/A_1_ ratio for the resonance polarization TM and for the core-shell emitters is close to 0.5 as it is obtained in theory for the average signal.

## Conclusions

We conclude that the photoluminescence of the dye molecules has two components when coupled to the grating nanostructure, a faster super-radiance followed by slower sub-radiance. Both times can be shorter than normal emitter. What is important is that the short pulse must have and has higher amplitude at the exponent. So short life time with high amplitude – these two properties cannot be separated. Indeed, the subradiant modes can be excited only through the coupling mechanism, sharing population from the superradiant modes. Thus the subradiant decay time is always longer and the amplitude is always less. Due to such an intrinsic link between two regimes the collective modes are always distinct and shown up in the form of fast and slow exponential decay. Averaging over many groups of emitters coupled with different coupling parameter G preserves the bi-exponential behavior of the decay with almost the same ratio ${\tau }_{2}/{\tau }_{1}\approx 5.5$ as the local ratio at specific G. This makes a strong evidence of the intrinsic exclusive properties of the coupling in each group of the coupled emitters. The counterintuitive result of our theory is confirmed by the experiments, that the amplitudes of two exponents, *A*_1_ and *A*_2_ for the average decay do not even cross the curves at any coupling parameter in the range. A high *A*_2_*/A*_1_ ratio is close to 0.5 in the case of core-shell Au/SiO_2_/ATTO655 nanoparticles. As one can see from Table 2(b) for samples 6, 7, 8 all the parameters out of the range for particular coupling parameters, but in the range for the average data.

This is indicated by the bi-exponential nature of the decay for rhodamine 800 dye near the metamaterial structure due to the interference caused by the nanostructure. This bi-exponential decay was observed for two very different plasmonic systems, subwavelength gratings and core-shell nanoparticles. By the changing the vertical substructure in each strip that support a plasmon resonance, and consequently the resonance wavelength spectral difference with the emission maximum, we realize the different coupling parameters (decreasing if the spectral difference is increasing). Two polarizations of light are also a way to change the coupling parameter, while it is hard to characterize quantitatively. Nevertheless, the experiments show qualitative correspondence with the theory.

## Methods

**Figure 7:**
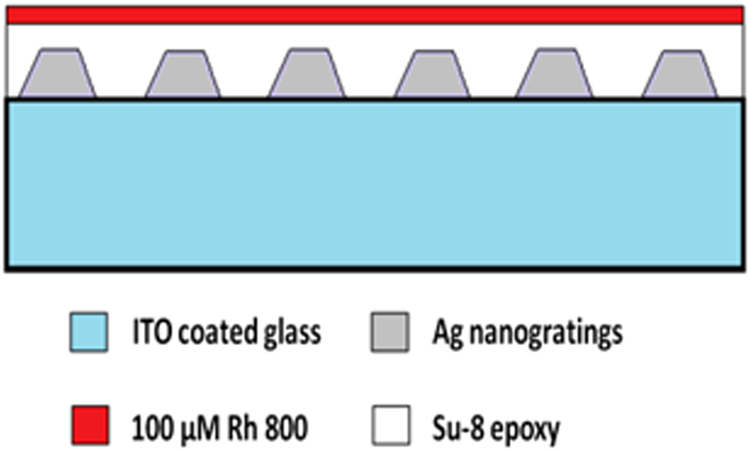
A grating with SU-8 epoxy spacer 28 nm, followed by Rh800 embedded in thinner epoxy 18 nm layer; gratings strips substructure: Ti 5 nm, 30 nm Ag, 40 nm alumina, 5 nm Ti, 30 nm Ag, and 10 nm alumina. The periodicity of the gratings is ~310 nm. The e-beam writing parameters were as follows: dose was 650, 725, 750, and 800 μC/cm^2^ for A, B, C, and D, respectively; 1.2 nA; and 100 kV accelerating voltage.

The paired Ag nanowire gratings schematically shown in Fig. 7 were fabricated by e-beam lithography. A photoresist layer was prepared by spin coating polymethyl methacrylate (950 PMMA A4, Microchem) onto a ITO coated glass substrate (Präzisions Glas & Optik, CEC500S) at 3000 rpm for 60 s, then baked for 60 minutes at 180 °C. A 100 nm gold frame was evaporated onto the substrate to calibrate the height of the sample for the Vistec VB6 electron beam (e-beam) writer. After exposure, the patterned was developed in MIBK:IPA 1:3 (Microchem) for 45 s followed by deposition of titanium (Ti) adhesion layer, silver (Ag), alumina (Al_2_O_3_), Ag, and Al_2_O_3_ as a protective layer with an e-beam evaporator at a rate of 0.5 Å/s for Ti and Ag and 1.0 Å/s for Al_2_O_3_. Liftoff was done in PG Remover (MicroChem) for 6 hours and rinsed with acetone after removal of PMMA photoresist. The structural characterization was done using a field emission scanning electron microscopy (FE-SEM), Hitachi S-4800.

Fluorescent lifetime image microscopy (FLIM) using the Picoquant Microtime 200 was done on paired Ag nanowire grating samples that had been spin coated with a 10% by volume SU-8 photoresist as a spacer layer (30 nm) then a 100 μM Rhodamine 800 dye in a 10% by volume SU-8 layer (22 nm). The life time imaging was done with a pulse diode laser (640 nm, 80 ps).

**Figure 8:**
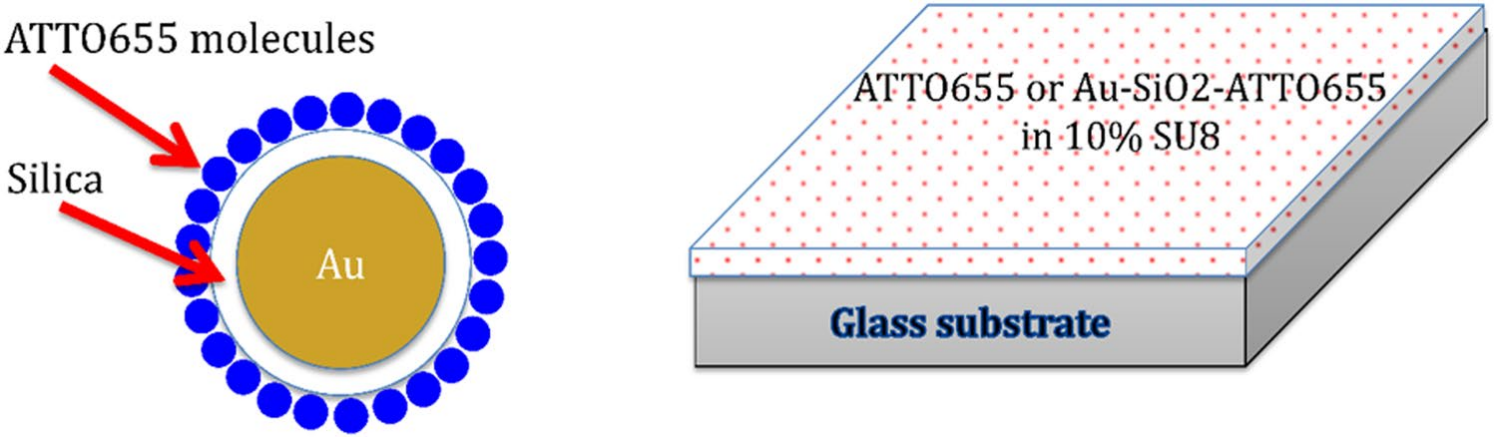
A core-shell particle: diagram, scheme of deposition on a glass substrate.

**Figure 9:**
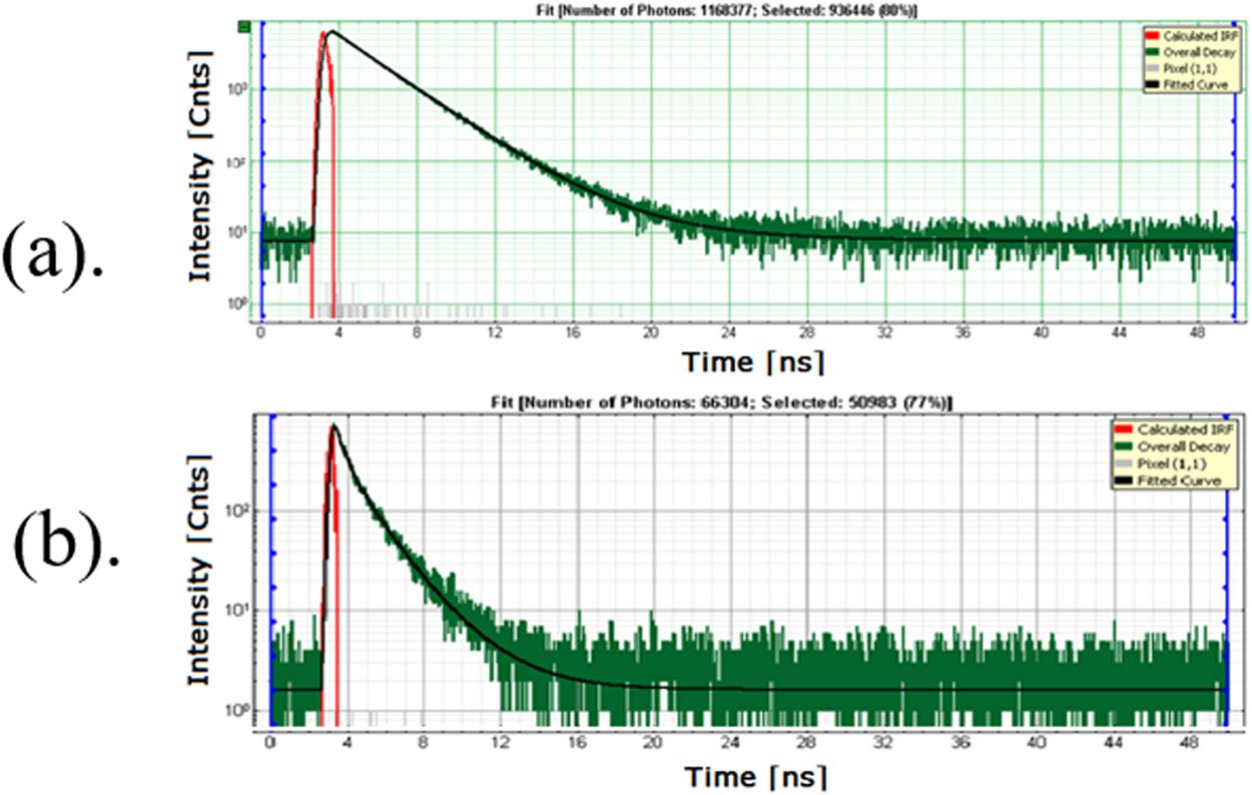
(**a**) A single exponential emission decay for Rh800 off the grating dies with life time 2.4 ns; (**b**) bi-exponential emission decay for Rh800 on the die; green- raw data, black – fitting with the exponential reconvolution, red –IRF.

Core-shell synthesis includes: tetraethyl orthosilicate (TEOS), ethanol, (3-aminopropyl) trimethoxysilane and ammonium hydroxide were purchased from Sigma-Aldrich (St. Louis, MO, USA). SU8-2000 epoxy is purchased from Micro Chem and Au 20 nm colloid from Ted Pella. ATTO655 is purchased from ATTO-TEC GmbH (Siegen, Germany). Synthesis of Au\SiO_2_ core-shell is done under vigorous stirring of 1 mL of Au 20 nm colloids concentration at 7 × 10^11^ particles/mL in 250 mL of 200 proof ethanol solution^13^. After the stirring was done for 10 minutes, 4 mL of 30% ammonium hydroxide was added. Immediately after adding ammonium hydroxide, 100 μL of TEOS were added to the solution. The solution was stirred for 2 hours and the solution aged without stirring at 4 °C for 24 hours. After that, solution was centrifuged (3000 rpm, 30 minutes) and washed with ethanol 4 times. Next, 500 μL of (3-aminopropyl) trimethoxysilane was added to the separated solution (10 mL) and stirred vigorously for 24 hours and separated again^13^. Preparation of fluorescent-coated Au\SiO_2_ core-shell is done by dyeing (doping) method. Add 4 mL of 6.2 μM ATTO655 (in ethanol) to 100 μL of Au\SiO_2_ (7 × 10^9^ particles) and keep it in a shaker for 24 hours. After completion of the procedure, once again separate the particles using previously mentioned method and re-dispersed in 10% SU8. Substrate preparation (see Fig. 8) for optical time resolved confocal fluorescence spectroscopy was done as follows. Thin substrates (0.25 mm thickness) were used in this experiment, and spin coated the final solution in 10% SU8 at different spin speeds from 500 rpm to 4000 rpm. Then we let it dry for optical measurements. The photoluminescence decay (Fig. 9) were fitted with exponential reconvolution (n = 3) using measured instrumental reference function (IRF, shown in red in Fig. 9):

$$I(t)={\int }_{-\infty }^{t}IRF(t\text{'})\sum _{i=1}^{n}{A}_{i}{e}^{-\frac{t-t\text{'}}{{\tau }_{i}}}dt\text{'}.$$

The fitting quality is characterized by the ${\chi }^{2}$ parameter, which is required to be as close to unity as possible. The Rh800 life time measured on the same substrate but without the Ag nanostructure is 2.4 ns and the decay is well fitted by the single exponential formula shown with black line in Fig. 9 (a). It is proven that the time dependence of photoluminesce for Rh800 (Fig. 9b) is bi-exponential, ${A}_{1}{e}^{-t/{\tau }_{1}}+{A}_{2}{e}^{-t/{\tau }_{2}}$, with a fast super-radiant mode and a slow subradiant emission. Fitting with three exponential terms gives the amplitude of third term negligible relative to the other two terms.

## Electronic supplementary material

Supplementary Information
