# Supplementary material for: Cooperative bi-exponential decay of dye emission coupled via plasmons
Source: Sci Rep. 2018 Jun 22;8:9508. doi: 10.1038/s41598-018-27901-4 (PMC6014996; doi:10.1038/s41598-018-27901-4)
Supplement: Supplementary file 1 — Supplementary Information [file 41598_2018_27901_MOESM1_ESM.pdf]

Supplementary Information

Cooperative bi-exponential decay of dye emission coupled via plasmons

David P. Lyvers[1,3], Mojtaba Moazzezi[1]*, Vashista C. de Silva[1], Dean P. Brown[3,4], Augustine M. Urbas[3], Yuri V. Rostovtsev[1], and Vladimir P. Drachev[1,2]†*

[1] Department of Physics and Advanced Materials Manufacturing Processing Institute, University of North Texas, Denton, TX 76203 USA.

[2]Skolkovo Institute of Science and Technology, Moscow, 121205, Russia.

[3]Air Force Research Laboratory, 3005 Hobson Way, Wright Patterson AFB, OH 45433, USA.

[4] UES, Inc., 4401 Dayton-Xenia Rd, Dayton, OH 45432, USA.

†vladimir.drachev@unt.edu

Structural characterization of the gratings

The structural characterization was done using a field emission scanning electron microscopy (FE-SEM), Hitachi S-4800. The experiments were performed with four metamagnetic gratings shown in Fig. SI1.

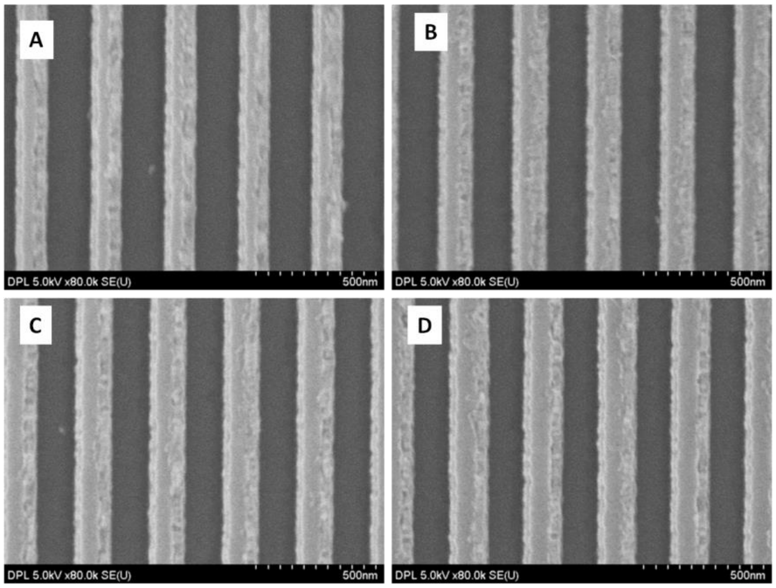

Figure SI1. Gratings strips substructure: Ti 5nm, 30 nm Ag, 40 nm alumina, 5 nm Ti, 30 nm Ag, and 10 nm alumina. The e-beam writing parameters were as follows: dose was 650, 725, 750, and 800 μC/cm^2 for A, B, C, and D, respectively; 1.2 nA; and 100 kV accelerating voltage. The periodicity of the gratings was ~310 nm. The bottom width of the trapezoidal gratings were 138.2 nm (±6.5 nm), 156.6 nm (±7.1 nm), 154.4 nm (±8.0 nm), and 168.9 nm (±7.0 nm) for A, B, C, and D, respectively. The top width of the trapezoidal gratings were 52.7 nm (±4.5 nm), 65.4 nm (±7.5 nm), 69.8 nm (±8.1 nm), and 84.0 nm (±7.6 nm) for A, B, C, and D, respectively.

Structural characterization of the core-shell synthesis

Fig. SI2 shows SEM images and characteristic dimensions of the core-shells. A variation in core-shell nanoparticle size and shape is shown here in Fig. SI2. A large scale SEM image in Fig. SI3 illustrate the core-shells distribution after deposition on a Si substrate.

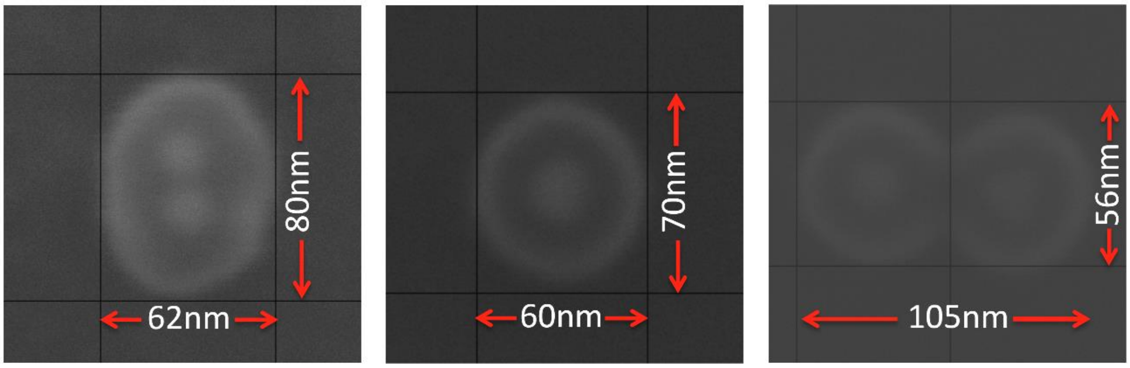

Figure SI2. Left image shows that it has a dimer core (two Au nanoparticles) inside the shell, which makes the "Y" axis longer than others. Middle image shows bit larger core-shell particle compared to shown in the main text in Figure 3a, which was the norm of the core-shell size we targeted, about 40 nm. Right image shows different conjoint core-shell particles, different between this and the left core-shell structure is that core particles are separated.

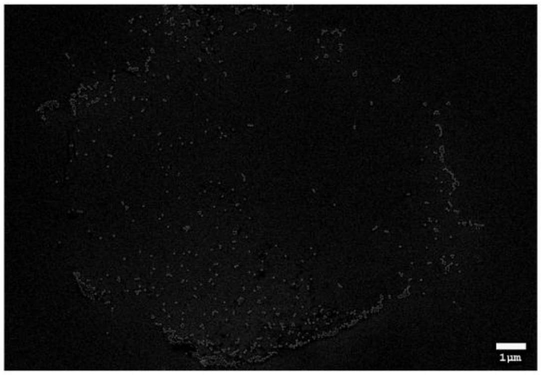

Figure SI 3. FE SEM image made on Si substrate scale bar 1 µm.

Gratings with Rh800

The fabrication of uniform gratings produced electric resonance around 500 to 525 nm and magnetic resonance spanning 650 nm to 800 nm. The magnetic resonance of the gratings are excellent candidates to affect the behavior of the Rh800 dye. As the width of the gratings is increased the magnetic and electric resonance red shifts to higher wavelength with TM illumination, the gratings behave as a dilute metal for TE polarization. The experimental spectra were matched using spatial harmonic analysis.[1]

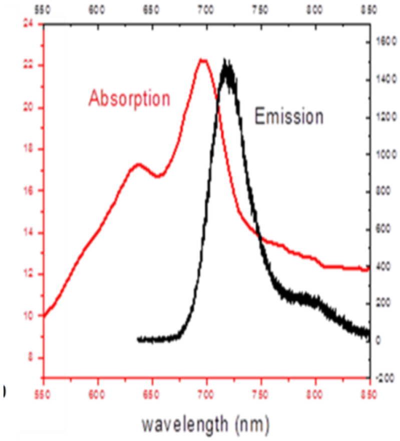

Figure SI3. Absorption and emission spectra of Rh800 (0.5 mM in 39 nm SU8 film).

The matching was done by optimizing the loss factor of the metals (Ti and Ag) and the refractive index of the alumina. The refractive index of the alumina is used as a tuning parameter since the optical properties could be inhomogeneous due e-beam evaporation and the refractive index has a range due to inhomogeneity of the evaporation process and crystallinity.[2]

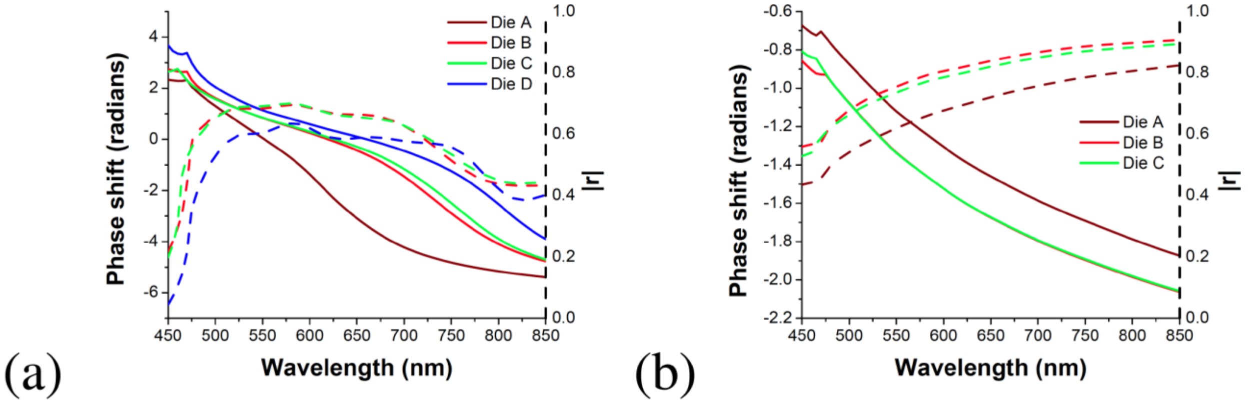

Figure SI4. (*a*) Amplitude reflection coefficient |r| (dashed lines) and phase shift (solid lines) for TM polarization dies A, B, C, D. (*b*) Amplitude reflection coefficient (dashed lines) and phase shift (solid lines) for TE polarization for dies A, B, C.

Figure SI4 shows the parameters retrieved for the effect layer for the phase shift and reflection amplitude.

References

1. Ni X. et al. PhotonicsSHA-2D: modeling of single-period multilayer optical gratings and metamaterials," https://nanohub.org/resources/sha2d. (DOI: 10.4231/D3WS8HK4X) (2012).

2. Eriksson, T. S., Hjortsberg, A., Niklasson, G. A. & Granqvist, C. G. Infrared optical properties of evaporated alumina films. *Appl. Opt.* **20,** 2742-2746 (1981).
